# WEIGHT LOSS AND THE LENGTH OF THE SMALL INTESTINE IN THE FOBI-CAPELLA SURGERY: IS THERE A RELATIONSHIP?

**DOI:** 10.1590/0102-672020210002e1634

**Published:** 2022-01-31

**Authors:** Oona Tomiê DARONCH, Hugo Genki Kagawa AKAHANE, Solange dos Anjos Cravo BETTINI

**Affiliations:** 1 Hospital de Clínicas - Universidade Federal do Paraná - PR, Cirurgia Geral - Curitiba - Paraná - Brasil

**Keywords:** Bariatric Surgery, Anastomosis, Roux-en-Y, Weight Loss, Cirurgia Bariátrica, Anastomose em - Y de Roux, Perda de Peso

## Abstract

Factors related to weight loss in obese patients undergoing bariatric surgery have always been exhaustively studied in an attempt to propose the best surgical technique with greater weight loss and long-term resolution of comorbidities. Patients present anatomical variations regarding the length of the small intestine. Some studies demonstrate weight changes in patients with different lengths of the intestinal loops in the Roux-en-Y bypass technique. The present work carried out a study on the influence of body mass index, weight loss, and common loop length on long-term surgical outcomes.

**METHODS:**

This is a descriptive cross-sectional study by retrospective analysis of 112 medical records of patients undergoing open bariatric surgery using the gastric bypass technique at University Hospital - UFPR. The data were correlated in statistical programs for this purpose.

**RESULTS:**

Out of 112 patients, 83.03% were women, with mean age of 41.52 years. The mean length of the total small bowel of the patients was 5.02 m. There was a directly proportional relationship between the length of the small intestine and weight loss (p=0.0428).

**CONCLUSION:**

There is a wide range of variables related to weight loss in patients undergoing bariatric surgery, such as the technique used, the length of the loops in the Roux-en-Y gastric bypass, and the routine of nutritional and physical monitoring of the patient. It is important to assess the technical details of the surgical procedure and to verify the weight loss by evaluating integrally the patient and other variables.

## RESUMO - RACIONAL

Os fatores relacionados à perda de peso nos pacientes obesos submetidos à cirurgia bariátrica sempre foram exaustivamente estudados na tentativa de propor a melhor técnica cirúrgica com maior perda de peso e resolução as comorbidades à longo prazo. Os pacientes apresentam variações anatômicas no que tange o comprimento do intestino delgado. Há estudos que demonstram alterações de peso nos pacientes que apresentam diferentes comprimentos das alças intestinais na técnica do by-pass em Y de Roux. O presente trabalho realizou um estudo entre a influência do IMC, a perda de peso e o comprimento da alça comum nos resultados cirúrgicos à longo prazo.

**MÉTODOS:**

Estudo transversal descritivo pela análise retrospectiva de 112 prontuários de pacientes submetidos à cirurgia bariátrica aberta pela técnica do bypass gástrico no Hospital de Clínicas -UFPR. Os dados foram correlacionados em programas estatísticos para este fim.

**RESULTADOS:**

Dos 112 pacientes, 83,03% eram do sexo feminino, média de idade de 41,52 anos. O comprimento médio do intestino delgado total dos pacientes foi de 5,02 metros. Houve uma relação diretamente proporcional entre o comprimento do intestino delgado e a perda de peso (p=0,0428).

**CONCLUSÃO:**

Há uma ampla gama de variáveis relacionadas à perda de peso nos pacientes submetidos à cirurgia bariátrica, tais como a técnica utilizada, o comprimento das alças no *by-pass* gástrico em Y de Roux e a rotina de acompanhamento nutricional e físico do paciente. É importante considerar os detalhes técnicos do procedimento cirúrgico, e verificar a perda de peso avaliando-se o paciente como um todo e outras variáveis.

## INTRODUCTION

Bariatric surgery is one of the main weight loss strategies in patients who are unable to achieve success with clinical treatment and change in lifestyle habits^5^. Several variables influence weight loss, as it is known that different individuals with similar body mass index (BMI), even when undergoing the same technique of bariatric surgery, have different results in long-term weight loss^5^.

The traditional indication for bariatric surgery in our literature are BMI values being >35 or >40 kg/m^2^ in patients with comorbidities. Factors related to weight loss in obese patients undergoing bariatric surgery have always been extensively studied in an attempt to propose the best surgical technique with greater weight loss and long-term resolution of comorbidities. It is known that patients have anatomical variations in the length of the small intestine, which varies in length from 3.36 to 7.64 m and has a diameter of approximately 4 cm, representing approximately an area of 250 m² ^2^ ^,^ ^6^ ^,^ ^7^.

Although individual metabolic, genetic, and lifestyle factors are intensively studied, it should be noted that patients have different lengths of the small intestine, and that this fact could be associated with weight loss, considering that the small intestine is responsible for the absorption of different nutrients. In addition, in the Fobi-Capella surgical technique, one of the most used techniques in our country, different surgeons use different lengths of alimentary limb (Roux limb) and biliopancreatic limb, raising the hypothesis that these lengths can be triggered in weight loss^3^ ^,^ ^10^ ^,^ ^12^.

In view of these different factors that can influence weight loss and improvement of the main comorbidities of patients over time, the objective of this study is to elucidate the factors that can be detached in performing bariatric surgery in different patient profiles.

## METHODS

This is a descriptive cross-sectional study, carried out through the retrospective analysis of patients undergoing open bariatric surgery using the Roux-en-Y gastric bypass (RYGB) technique at a tertiary hospital in southern Brazil between June 2013 and December 2019, analyzing 118 patients. There were considered data referring to the preoperative and postoperative BMI at 6 and 12 months, and the length of the intestinal loops in the preoperative period, present in the surgical description, described during the intraoperative period before performing the Roux-en-Y technique.

The results obtained were quantified in a spreadsheet using Google Docs. Statistical data were calculated using specific programs for this purpose (SPSS 2.0). The value of p<0.05 was considered statistically significant.

The inclusion criteria were patients undergoing open bariatric surgery using RYGB from June 2013 to December 2018, who presented a complete medical record and the preoperative and postoperative BMI values after 6 and 12 months of surgery. In addition, there were included patients who had a complete surgical description referring to intraoperative data on the total length of the small bowel.

Exclusion criteria of patients were below 18 years old, presence of medical records with incomplete data, absence of surgical description or absence of data related to the length of the small loops, irregular follow-up in the postoperative period, and absence of preoperative and postoperative BMI in medical records. Out of 118 patients who underwent bariatric surgery during the study period, 6 patients were excluded: 2 of whom underwent sleeve technique, and 4 had medical records with incomplete data about weight loss at 6 and 12 months or about the total length of the small intestine. Thus, 112 patients were included in this study. This study was approved by the Research Ethics Committee (CEP) of the hospital under protocol number 36482620.2.0000.0096 (from CAAE).

## RESULTS

In this study, out of 112 patients diagnosed with bariatric surgery using RYGB, 93 patients (83.03%) were women and 19 patients (16.97%) were men. The mean age was 41.52 years, with a standard deviation of 12.22. The mean weight, height, and BMI were 114.3 kg, 1.61 m, and 44.08 kg/m^2^, respectively.

The mean length of the total small intestine of the patients was 5.02 m, the shortest length found was 4 m, and the longest length was 6.7 m. In women, the average length was 5.02 m, and in men, the average length was 5.05 m.

The results indicated above, as well as the mean length and standard deviation of the alimentary, biliopancreatic, and common loops are represented in Table 1.

**Table 1 -:** Profile of the study sample (n=112).

	M (n=19)	F (n=93)	Total (n=112)
Age	38.74(±5.55)	42.09(±10.88)	41.52(±12.22)
Height	1.71(±0.06)	1.59(±0.07)	1.61(±0.09)
Small intestine	5.05(±0.3)	5.02(±0.68)	5.02(±0.74)
Biliopancreatic loop	1(±0)	0.99(±0.05)	0.99(±0.05)
Alimentary loop	1(±0)	1.01(±0.05)	1.01(±0.05)
Loop	3(±0.3)	3.02(±0.66)	3.02(±0.72)
Weight	130.89(±10)	110.91(±16.76)	114.3(±19.52)
BMI	44.81(±1.99)	43.93(±5.57)	44.08(±5.91)

Regarding the postoperative follow-up of patients at 6 and 12 months, it was found that the average weight loss in the first semester, considering the entire study sample, was 27.1 kg, with an average loss of 10.4 points in BMI in that period. In the second semester (in 12 months), the average weight loss was 35.7 kg with an average loss of 13.8 points in BMI. These results are given in Table 2.

**Table 2 -:** Weight loss and BMI follow-up at 6 and 12 months.

Average (±DP)		Weight	Weight loss	BMI	BMI loss
Follow-up (semesters)	0	114.3(±19.5)	-	44.1(±5.9)	-
	1	87.1(±16.6)	27.1(±9.9)	33.6(±5.3)	10.4(±3.6)
	2	77.6(±15.2)	35.7(±11.4)	30.1(±4.9)	13.8(±4.1)

Observing the female and male genders individually, there was a greater weight loss in male patients, with a mean loss of 30.9 kg in the 6-month follow-up and 40 kg in the 12-month follow-up. In contrast, in female patients, there was an average weight loss of 26.3 kg in 6 months and 34.8 kg in 12 months. However, the initial weight of men was higher (mean of 130.9 kg) than that of women (mean of 110.9 kg) preoperatively. These results, together with data of loss of BMI over the period studied, are given in Table 3.

**Table 3 -:** Weight loss and BMI at 6 and 12 months follow-up separated by gender.

Average(±DP)		Total (M)			
		Weight	Weight loss	BMI	BMI loss
Follow-up (semesters)	0	130.9(±10)	-	44.8(±2)	-
	1	100(±8.7)	30.9(±4.6)	34.3(±2.2)	10.5(±1.4)
	2	91(±8.1)	40(±5.4)	31.3(±1.9)	13.8(±1.7)
Average (±DP)		Total (F)			
		Weight	Weight loss	BMI	BMI loss
Follow-up (semesters)	0	110.9(±16.8)	-	43.9(±5.6)	-
	1	84.4(±14.1)	26.3(±8.7)	33.5(±4.9)	10.4(±3.4)
	2	74.9(±12.8)	34.8(±10)	29.8(±4.6)	13.8(±3.7)

**Figure 1 -:**
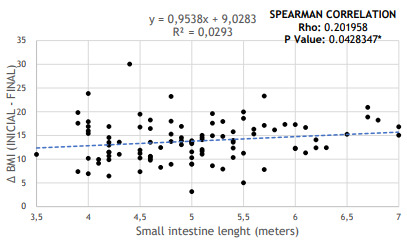
Relationship between small intestine length and weight loss, assessed by the difference between final and initial BMI (p=0.0428).

When performing a correlation between weight loss and the initial length of the small intestine, it was possible to observe that patients who dissipated greater weight loss (greater difference in BMI before bariatric surgery and in the 12-month follow-up) had longer total bowel length. Thus, there was a directly proportional relationship between the length of the small intestine and weight loss, with a p-value of 0.0428 (p<0.05). Figure 1 shows this correlation.

**Figure 2 -:**
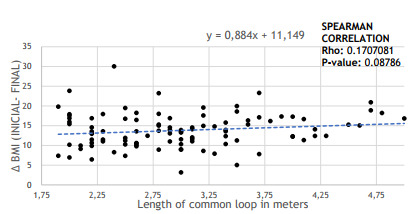
Relationship between common loop length and weight loss, assessed by the difference between final and initial BMI (p=0.08786).

When performing a correlation between the length of the common loop and the loss of BMI (assessed by the difference between the BMIs in the preoperative period and in the 12-month follow-up), there was no statistical difference (p=0.087 and p>0.05). Therefore, it was not possible to state in this study that patients with small bowel size in the common loop have greater weight loss and greater variation in BMI. This correlation is illustrated in Figure 2.

## DISCUSSION

The small intestine is responsible for the absorption of most nutrients and vitamins, such as vitamin D, iron, and folic acid, for the proper functioning of the body and for producing hormone GLP-1 and peptide YY, which are essential for gastric emptying of satiety and delay in gastric emptying, the former also contributing to the stimulation of insulin release^6^.

The understanding of the complex mechanism of intestinal absorption and the perception that different lengths of small intestine are part of them, from the angle of Treitz to the ileocecal valve, led to the emergence of different hypotheses related to the mechanism of obesity, proposing that participating than presenting greater intestinal length presents greater nutritional absorption and, consequently, a greater tendency toward obesity^6^, according to the long intestine hypothesis. The present study showed a directly proportional relationship between total small bowel length and weight loss, with a statistical p-value for disease (p=0.042).

Thus, several studies have been and are being carried out with the aim of better understanding the factors involved in the pathophysiology of obesity. Dietary limb length and function have been studied extensively, but few studies have studied the influences of biliopancreatic limb length^9^.

It is known that the small intestine varies in length from 3.36 to 7.64 m, has a diameter of approximately 4 cm, and represents approximately an area of 250 m² (considering a person of 1.70 m) for nutrient absorption^1^. In this study, it was possible to observe an average length of 5.02 m when considering both sexes. It is believed that weight loss after biliopancreatic diversion and duodenal switch is inversely related to the length of the alimentary limb and common channel. However, the effect of the length of the biliopancreatic limb (GLP-1) on weight loss has less attention^3^.

A previous study carried out in 2009 at a University Hospital in Curitiba, with 30 patients who underwent gastroplasty using the Fobi-Capella technique (RYGB), showed that the mean intestinal length for men and women was 582.5 and 509.1 cm, respectively (an average intestinal size of 528.7 cm for the entire sample). From the calculation of Pearson’s correlation coefficient, in this study, the absence of correlation between BMI and small bowel length was confirmed^6^. Similarly, the present study showed that the average total length of the small intestine in women and men was 5.02 and 5.05 m, respectively. In contrast, it was possible to verify that there was no correlation between weight loss assessed by BMI and the total length of the small intestine. The expected results were not satisfactory in patients who underwent the Fobi-Capella technique and who received other techniques for reoperation with the aim of achieving greater weight loss. The options were the Fobi, Brolin, or distal gastrojejunoileal (Scopinaro type) techniques. First, the enteroenteric anastomosis is removed and then distally remade in half the length of the small intestine, which is again measured from the angle of Treitz^7^. Thus, the common canal and the alimentary canal are of 3-3.5 m, the latter remaining 90 cm. In the Brolin-type technique, the enteroenteric anastomosis is not performed, which is remade distally, 75 cm from the ileocecal valve, thus leaving the common canal with 75 cm in length, and in addition to the excluded biliopancreatic loop (BPL), with 30 cm, the remainder of small intestine being stabilized as alimentary canal. In the Scopinaro technique, the enteroenteric anastomosis is not performed, which is remade with an afferent jejunal loop and the ileal loop of 100 cm from the ileocecal valve^7^. The jejunal part of the alimentary loop is resected, and an enteroenteric anastomosis is performed between the ileum (220 cm) loop and the remainder of the jejunal loop, on average 20 cm, which is anastomosed to the gastric pouch. Thus, an alimentary canal is composed on average of 20 cm of jejunum and 220-230 cm of ileum^7^. In our study, however, it was not possible to verify a statistically significant association between the length of the common loop and weight loss.

A study carried out with 41 patients, 32 of whom underwent reoperation by one of the three surgical techniques (i.e., Fobi, Brolin, and distal gastrojejunoileal bypass) showed a decrease in the absorptive area of the small intestine^7^. Among these, the one that showed results superior to others in terms of weight loss was the technique of distal gastrojejunoileal bypass (69.7%). This result possibly occurs because this technique increases discomfort and, consequently, weight loss but increases the rate of nutritional complications. In the present study, we only performed a comparison between patients undergoing gastric bypass.

The factor that certainly presents a positive association between the length of the small loops was height^11^. The factors that interfere with weight loss and resolution of metabolic comorbidities are gender, age, height, and expected jejunal length^11^. Thus, measuring the length of the small loops can prevent the risk of nutritional consequences in malabsorptive, revisional, and metabolic procedures. In this analysis, for any height, with longer expected jejunal length, patients will be more obese and, for any expected jejunal length, taller patients will have a lower weight^11^.

A study with a total of 1001 patients after biliopancreatic/duodenal diversion (209 men and 792 women, mean age 42±10 years, mean BMI 52±9 kg/m^2^) was divided into two groups, according to the ratio of the length of the biliopancreatic limb (GLP-1) to the total length of the small intestine (BLS): a GLP-1 ≤45% of the SBL versus a GLP-1 >45% of the SBL^3^. Biliopancreatic diversion/duodenal switch is directly related to the proportion of small bowel diverted in patients with BMI >60 kg/m^2^. Furthermore, the effect increased with the duration of follow-up^3^.

The length of the common canal may also be important in weight loss after biliopancreatic diversion/duodenal switch, as shown in the study by Hamoui *et al.* ^3^, in which patients with a 100 cm common canal lost more weight than those with a common canal of 150 cm. In the present study, it was not possible to carry out this correlation, as the standardization of the routine in our hospital is to perform a food loop and a BPL with 100 cm each, and the common loop is variable according to the total length of the patient’s thin preoperatively.

Many projects were carried out to identify the ideal length of the alimentary limb capable of providing greater and sustainable weight loss with fewer comorbidities (mainly nutritional), but so far there is no consensus^12^. In a literature review of 13 papers, it was observed that the release of enterohormones in response to a food load in the distal small intestine plays an important role in the remission of comorbidities. Therefore, the length of the BPL can affect this process. Gastric restriction combined with a modest degree of deviation of the BPL resulted in a complete postoperative weight loss compared with the conventional Roux-en-Y bypass with lengths of 15 cm loop for the BPL and 75 cm for the alimentary loop^12^.

In addition to greater weight loss, increased length of the BPL could be related to metabolic improvement in diabetes. The largest series comparing different loop lengths with more than 500 patients found no difference in HbA1c in diabetic patients between a long loop (200 cm) and a classic RYGB; however, long BPL RYGB was associated with a significant decrease in HbA1c compared with classic RYGB in non-diabetic patients^12^.

It is known that, in super-obese patients, the rates of weight loss and weight recovery are high after the Roux-en-Y deviation, and another study revealed that to improve weight loss, it is necessary to stretch the biliopancreatic limb^10^. A retrospective cohort of 671 super-obese patients operated over a period of 10 years was carried out. Patients were classified into three groups: (1) 155 patients with a common loop of 150 cm and a BPL of 60 cm; (2) 230 patients with a common loop of 60 cm and a BPL of 200 cm; and (3) 286 patients with a common loop of 150 cm and a BPL of 200 cm. The total length of the BPL was shortened to 60 cm in group 1 and 200 cm in groups 2 and 3. When comparing three groups, it should be noted that a weight loss failure was greater in group 1 (10.3%) compared with the other groups (4.3%; 5.2%). Group 3 had minor secondary weight regain (26.6%). The remission of comorbidities was greater in the groups with 2 m of BPL at the expense of nutritional and vitamin deficiencies (3.9%; 5.9%). There was no difference in hypoalbuminemia^10^.

Recognizing that weight loss was similar in the 200-cm-long groups, another study revealed that the size of the alimentary limb is extremely important for long-term weight loss, rather than focusing primarily on common canal shortening^10^. Thus, it was proposed to shorten the alimentary limb with a BPL of 200 cm and a common loop of 100 cm. Given that the total length of the small intestine varies between people, the work eliminated that reducing the length of the food loop by one-third - instead of a fixed number - is particularly important in cases of very short length of the small intestine in order to avoid malnutrition and malabsorption^10^. In this study, no statistically summarized relationship (p>0.05) was found between the size of the common loop and weight loss. The comparison between the size of the food loop and weight loss was not performed, as the food loop is standardized around 100 cm in the routine of our service, as previously mentioned.

Most published studies do not consider the influence of the common loop on weight loss but only the alimentary loop and BPL. The study carried out in Spain^1^ with 151 patients diagnosed with videolaparoscopic bariatric surgery using RYGB showed that a common loop has no effect on weight loss in patients who underwent RYGB and that a reduced length of the common loop is related to greater nutritional deficiencies. Based on BMI, patients were divided into two groups as follows: (1) morbidly obese (BMI 35-50 kg/m^2^) with 115 patients and (2) morbidly obese and obese (BMI >50 kg/m^2^) with 36 patients. The length of the biliopancreatic limb was 100 cm in both groups; the feeding member was 150 cm in the obese group (first group) and 200 cm in the super obese group (second group). The 50% common loop percentage is statistically associated with iron, ferritin, and protein (total protein and albumin) deficiencies. Therefore, more rigorous nutritional blood tests must be performed to provide early treatment with supplements and a correct approach to the patient. However, considering that the percentage of bowel used in the common limb does not influence the percentage of excess weight loss (EWL) in obese or super-obese patients who undergo laparoscopic RYGB, there is no indication in changing the length of the common loop to weight loss. Similarly, in this series, it was also not possible to make a significant association between the length of the common loop and weight loss (p=0.087 and p>0.05).

Regarding the variation in the length of the food loop and BPL and the improvement in metabolic parameters (e.g., DM, SAH, dyslipidemia, and abdominal circumference), studies show that there was no relationship between the variables^8^. This study was carried out in 63 patients in a retrospective cohort, and the patients were divided into three groups as follows: group 1: a BPL of 50 cm and a food loop of 100 cm; group 2: a BPL of 50 cm and a food loop of 150 cm; and group 3: a BPL of 100 cm and a food loop of 150 cm. When comparing the groups, there was also no statistical difference in weight loss between the groups, and the waist circumference measurements were homogeneously reduced in all groups^8^.

Comparing groups with different lengths of alimentary loop and BPL, other authors demonstrated differences in the control of DM2 by analyzing two groups with different length loops (group 1: a BPL of 50 cm and a food loop of 150 cm; and group 2: a BPL of 100 cm and alimentary loop of 250 cm). Diabetes was controlled in 58% of group 1 and in 93% of group 2 (p<0.05). Lipid disturbances improved in 57% of group 1 and in 70% of group 2 (p<0.05). No statistical difference was found in the control or improvement of hypertension, sleep apnea, or gastroesophageal reflux disorder. The loss of excess weight was faster in group 1 but was similar in both groups at 48 months (70% in group 1 and 74% in group 2), with no statistical difference. Those with longer intestinal deviations had better DM2 control (p<0.05)^8^.

A failure of RYGB surgery can be defined as weight loss failure, i.e., excess weight loss <50% or BMI >35 kg/m^2^, whereas weight gain occurs in up to 35% of patients^4^. There is no consensus regarding the best technique for reviewing the initial surgery. One of the options is the conversion of RYGB to a long BPL, and the results in the short term are promising. Research was carried out in 28 patients for revision surgery, with lengths of 150 cm (95% CI 133-156 cm) for the BPL and 100 cm (95% CI 97-113 cm) for the alimentary limb, thus providing a median total length of 250 cm^4^. The technical principle for achieving greater weight loss was the shortening of the total length of the alimentary limb (and the exclusion of a greater amount of small intestine in the BPL)^4^. This surgery resulted in an additional reduction in BMI of 10.0 kg/m^2^; however, a high rate of protein-calorie malnutrition with a common length of 250 cm raises great concerns regarding the general practice and safety of this malabsorptive revisional technique, with six patients requiring reoperation due to severe malnutrition of this technique^10^. Therefore, it is not recommended to reduce the length of the alimentary loop in patients who are at risk of malnutrition, which is why we have maintained the standard 100 cm for this loop in our service.

Finally, the literature shows a wide range of variables related to weight loss in patients undergoing bariatric surgery, such as the technique used, the length of the loops in the RYGB, and also the routine of nutritional and physical monitoring of the patient.

## CONCLUSION

It was possible to verify a directly proportional relationship between the length of the small intestine and weight loss, but no statistically significant correlation was obtained between the length of the common loop and weight loss, showing that possibly only the lengths of the alimentary loop and BPL have an entry into weight loss, according to other studies. Therefore, it is important not only to assess the technical details of the surgical procedure but also to verify weight loss by evaluating the patient as a whole and the other variables present.

## References

[1] Abellan I, Luján J, Frutos MD, Abrisqueta J, Hernández Q, López V, Parrilla P (2014). The influence of the percentage of the common limb in weight loss
and nutritional alterations after laparoscopic gastric
bypass. Surg Obes Relat Dis.

[2] De Oliveira GJM, Schieferdecker MEM, Campos ACL (2021). Are enterotypes in obese modified by bariatric surgery, the use
of probiotic supplements and food habits?. Arq Bras Cir Dig.

[3] Hamoui N, Anthone GJ, Kaufman HS, Crookes PF (2008). Maintenance of weight loss in patients with body mass index
>60 kg/m2: importance of length of small bowel bypassed. Surg Obes Relat Dis.

[4] Kraljević M, Köstler T, Süsstrunk J, Lazaridis II, Taheri A, Zingg U, Delko T (2020). Revisional Surgery for Insufficient Loss or Regain of Weight
After Roux-en-Y Gastric Bypass: Biliopancreatic Limb Length
Matters. Obes Surg.

[5] Lucas RWDC, Nassif PAN, Tabushi FI, Nassif DSB, Ariede BL, Brites-Neto J, Malafaia O (2020). Can stature, abdominal perimeter and BMI index predict possible
cardiometabolic risks in future obesity?. Arq Bras Cir Dig.

[6] Nassif PAN, Malafaia O, Ribas CAPM, Pachnicki JPA, Kume MH, Macedo L (2009). Correlation study of BMI and small intestine length in obese
patients subjected to bariatric surgery. Arq Bras Cir Dig.

[7] Pareja JC, Pilla VF, Callejas-Neto F, Coelho-Neto Jde S, Chaim EA, Magro DO (2005). Gastric bypass Roux-en-Y gastrojejunostomy--conversion to distal
gastrojejunoileostomy for weight loss failure - experience in 41
patients. Arq Gastroenterol.

[8] Pinheiro JS, Schiavon CA, Pereira PB, Correa JL, Noujaim P, Cohen R (2008). Long-long limb Roux-en-Y gastric bypass is more efficacious in
treatment of type 2 diabetes and lipid disorders in super-obese
patients. Surg Obes Relat Dis.

[9] Ramos RJ, Mottin CC, Alves LB, Benzano D, Padoin AV (2016). Effect of size of intestinal diversions in obese patients with
metabolic syndrome submitted to gastric bypass. Arq Bras Cir Dig.

[10] Shah K, Nergård BJ, Fagerland MW, Gislason H (2019). Limb Length in Gastric Bypass in Super-Obese Patients-Importance
of Length of Total Alimentary Small Bowel Tract. Obes Surg.

[11] Tacchino RM (2015). Bowel length: measurement, predictors, and impact on bariatric
and metabolic surgery. Surg Obes Relat Dis.

[12] Zorrilla-Nunez LF, Campbell A, Giambartolomei G, Lo Menzo E, Szomstein S, Rosenthal RJ (2019). The importance of the biliopancreatic limb length in gastric
bypass: A systematic review. Surg Obes Relat Dis.

